# IL-6-Producing Pheochromocytoma Associated With Von Hippel Lindau Disease: A Case Report With Literature Review

**DOI:** 10.7759/cureus.52760

**Published:** 2024-01-22

**Authors:** S Hata, Mayuka Shinohara, Tadasuke Ando, Hiromitsu Mimata, Toshitaka Shin

**Affiliations:** 1 Department of Urology, Faculty of Medicine, Oita University, Yufu, JPN

**Keywords:** stat3, von hippel lindau disease, adrenalectomy, pheochromocytoma, interleukin-6

## Abstract

We present a first case report of an IL-6-producing pheochromocytoma associated with von Hippel Lindau (vHL) disease. Pheochromocytomas are rare tumors that produce catecholamines, leading to various symptoms. In this case, a 28-year-old woman with a family history of vHL disease presented with a prolonged fever. Laboratory examinations revealed elevated C-reactive protein levels, and notably, a significantly increased serum IL-6 level. Imaging studies confirmed bilateral adrenal tumors with increased uptake on fluorodeoxyglucose-positron emission tomography and ^123^I-metaiodobenzylguanidine scintigraphy in the right adrenal gland. Despite partial relief with nonsteroidal anti-inflammatory drugs and alpha-blockers, her fever persisted until prednisolone administration, which promoted a complete resolution. A histopathological analysis following a right laparoscopic adrenalectomy revealed a typical pheochromocytoma. We conducted further analyses, including an enzyme-linked immunosorbent assay (ELISA), a quantitative real-time polymerase chain reaction (PCR) test, and immunoblot assays from the resected tumor tissues. We compared the current case with other cases of pheochromocytoma that presented neither elevated serum IL-6 nor high fever. Using ELISA, we found that this patient exhibited more IL-6 secretion than that seen in other cases. Additionally, quantitative real-time PCR and immunoblot found that both the phosphorylated signal transducer and activator of transcription 3 (STAT3) messenger RNA (mRNA) and protein expression levels exceeded those of the other cases. Thus, we surmised that IL-6 was produced directly from the tumor tissue and IL-6 expression was potentiated through the IL-6/STAT3 signaling pathway. Our findings contribute to the understanding of IL-6-producing pheochromocytomas and their distinct clinical characteristics.

## Introduction

Pheochromocytomas and paragangliomas (PPGLs) are tumors arising from chromaffin cells in the adrenal medulla or paraganglia that produce catecholamines. The excessive production of catecholamines by PPGLs produces varied symptoms including palpitations, sweating, pallor, headache, weight loss, chest pain, and distinctive hypertension patterns [1]. Fever is an uncommon manifestation of PPGLs. In a variety of tumors, tumor-produced IL-6 reportedly contributes to the occurrence of fever, and IL-6 governs the synthesis of acute-phase proteins, such as C-reactive proteins (CRPs) [2]. Only a few case reports for pheochromocytoma have been published to date in which fever was the chief complaint and excessive IL-6 production was ultimately attributed to the fever [3-5]. However, it is unclear by what mechanism the tumor overproduces IL-6 in pheochromocytomas. This report describes the first case of IL-6-producing pheochromocytoma associated with von Hippel Lindau (vHL) disease in a young woman. We further analyzed the mechanism by which tumors overproduce IL-6 using molecular biological techniques.

## Case presentation

**Figure 1:**
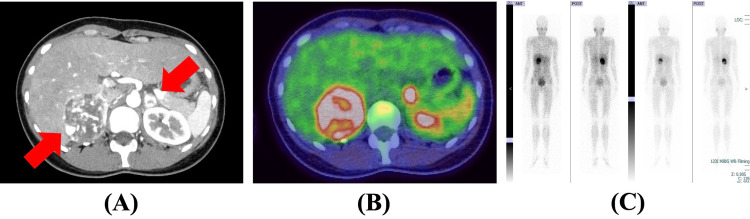
Imaging finding of bilateral adrenal tumors (A): A contrast-enhanced CT image showing bilateral adrenal tumors (arrow). (B): A fluorodeoxyglucose positron emission tomography image showing abnormal uptake on bilateral adrenal tumors. (C): ^123^I-metaiodobenzylguanidine scintigraphy showing increased uptake in the right adrenal gland.

A 28-year-old woman presented with a protracted fever that lasted >one month. She exhibited no symptoms related to the respiratory, gastrointestinal, or urinary systems. Her father and grandmother had undergone an adrenalectomy for pheochromocytoma diagnosis and had been diagnosed with vHL. However, she had not previously been surveilled for vHL. She had no previous medical history, and her menstrual cycle was regular. Both COVID-19 and influenza virus infections were ruled out. Although she was prescribed quinolone antibiotics and acetaminophen, the fever persisted. The complete blood count revealed a white blood cell count of 9.29 × 10^3^/μL, a red blood cell count of 4.23 × 10^6^/μL, a hemoglobin level of 11.3 g/dL, and a platelet count of 547 × 10^3^/μL. All biochemical analysis results were within the normal range, except for an exceptionally high CRP level of 30.6 mg/dL. A contrast abdominal CT scan was performed, showing bilateral adrenal tumors (Figure 1A).

Subsequently, the patient was referred to our hospital for a detailed examination and treatment. Upon re-examination (Table 1), the physical assessment revealed no additional abnormalities, but her body temperature was recorded as 39.2℃, and her blood pressure was 128/78 mmHg. The elevated CRP level persisted at 28.41 mg/dL. An endocrinological examination revealed urinary adrenaline of 7.9 μg/day, urinary noradrenaline of 1858.3 μg/day, urinary metanephrines of 0.12 mg/day, and urinary normetanephrines of 7.1 mg/day. IL-6 was elevated, and inflammatory response was elevated (Table 2).

**Table 1:** Laboratory data on admission INR, international normalized ratio; APTT, activated partial thromboplastin time; T-Bil, total bilirubin; AST, aspartate transaminase; ALT, alanine aminotransferase; ALP, alkaline phosphatase; GTP, glutamyl transpeptidase; LDH, lactate dehydrogenase; TP, total protein; Alb; albumin; BUN, blood urea nitrogen; Cr, creatinine; Hb, hemoglobin; CRP, C-reactive protein; TSH, thyroid stimulating hormone; FT4, free T4; sIL-2R, soluble interleukin-2 receptor; PAC, plasma aldosterone concentration; ARC, active renin concentration; DHEA-S, dehydroepiandrosterone sulfate; ACTH, adrenocorticotropic hormone; TNF, tumor necrosis factor.

Laboratory examination	Value	Unit	Reference range	Laboratory examination	Value	Unit	Reference range
Hematology				BUN	8.3	mg/dL	8ー20
RBCs	404	103/μL	386-492	Cr	0.7	mg/dL	0.46-0.79
Hemoglobin	10.4	g/dL	11.6-14.8	Glucose	103	mg/dL	73-109
WBCs	7800	/μL	3300-8600	HbA1c	6.2	%	4.9-6.0
Neutrophil	75.4	%	38.5-80.5	Na+	134.8	mEq/L	138-145
Lymphocyte	15.3	%	16.5-49.5	K+	4.57	mEq/L	3.6-4.8
Monocyte	9.2	%	2.0-10.0	Cl+	97	mEq/L	101-108
Eosinophil	0.0	%	0.0-8.5	CRP	28.41	mg/dL	0-0.14
Basophil	0.1	%	0.0-2.5	TSH	1.19	μIU/mL	0.5-5
Platelets	463	103/μL	158-348	FT4	0.85	ng/dL	0.9-1.7
Coagulation				sIL-2R	350	U/mL	204-587
Prothrombin time	16.0	second	9.9-11.8	PAC	123.6	pg/mL	35.7-240
INR	1.39		0.85-1.15	ARC	3.8	pg/mL	3.2-36.3
APTT	60.4	second	24-39	DHEA-S	103	μg/dL	19-231
Biochemistry				ACTH	6.42	pg/mL	7.2-63.3
T-Bil	0.35	mg/dL	0.4-1.5	Cortisol	22.0	μg/dL	7.07-19.6
AST	28.1	IU/L	13-30	procalcitonin	0.2	ng/mL	<0.05
ALT	21.6	IU/L	7ー23	TNF-α	6.88	pg/mL	2.27-11.2
ALP	107	IU/L	38-113	IL-6	76.8	pg/mL	<2.41
γ-GTP	19.7	IU/L	9-32	Urinalysis			
LDH	370	IU/L	124-222	Glucose	-		
TP	7.84	g/dL	6.6-8.1	Occult blood	1+		
Alb	2.98	g/dL	4.1-5.1	Protein	±		

**Table 2:** Laboratory data before and after right adrenalectomy CRP, C-reactive protein.

Laboratory examination	Value		Unit	Reference range
	pre-operation	post-operation		
Plasma				
IL-6	76.8	2.3	pg/mL	<2.41
CRP	28.41	0.11	mg/dL	0-0.14
Adrenaline	<0.01	<0.01	ng/mL	<0.17
Noradrenaline	7.2	1.1	ng/mL	0.15-0.57
Dopamine	0.06	<0.02	ng/mL	<0.03
Metanephrine	<13	19	pg/mL	<130
Normetanephrine	1620	148	pg/mL	<506
Urine				
Adrenaline	7.9	-	μg/day	3.0-41.0
Noradrenaline	1858.3	-	μg/day	31.0-160.0
Dopamine	1466.8	-	μg/day	280.0-1100.0
Metanephrine	0.12	-	mg/day	0.05-0.20
Normetanephrine	7.1	-	mg/day	0.10-0.28

**Figure 2:**
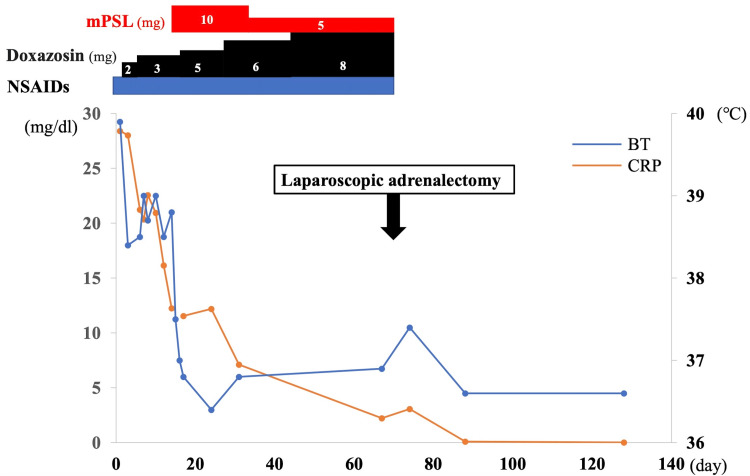
Body temperature (BT) and C-reactive protein (CRP) level before and after treatment mPSL, methylprednisolone pulse therapy; NSAIDs, nonsteroidal anti-inflammatory drugs.

Imaging studies showed increased uptake on fluorodeoxyglucose (FDG) positron emission tomography (PET), consistent with bilateral adrenal masses, (Figure 1B) and on ^123^I-metaiodobenzylguanidine (MIBG) scintigraphy in the right adrenal gland (Figure 1C). Based on the elevated catecholamine levels, the increased inflammatory response with a marked increase in IL-6, and her family history, we considered the adrenal masses to be IL-6-producing pheochromocytomas. After admission, nonsteroidal anti-inflammatory drugs (NSAIDs) (loxoprofen) and doxazosin were started, which tended to decrease the CRP level but not her fever. Therefore, prednisolone was started 15 days after admission, which subsequently promoted the complete resolution of her fever (Figure 2).

**Figure 3:**
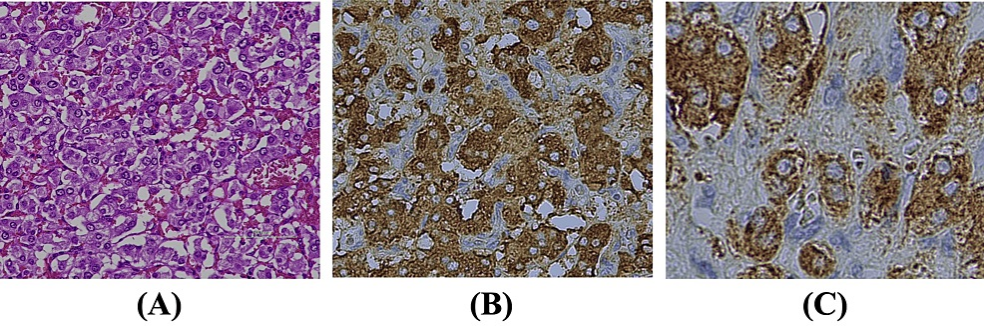
Immunostaining images of the tumor specimen (A): H&E staining of the resected tissue (×400). (B): Chromogranin A immunostaining of the resected tissue (×400). Tumor cells were focally positive for chromogranin A. (C): Synaptophysin immunostaining of the resected tissue (×400). Immunostaining showed positive results in the resected tissue.

After the patient showed an improved inflammatory response, a right laparoscopic adrenalectomy was performed. Because of tumor compression during the surgery, the systolic blood pressure increased to 200 mmHg, so calcium channel blocker therapy was initiated. Postoperatively, the levels of inflammatory markers improved, and also serum-free normetanephrine and IL-6 levels dramatically decreased (Table 2). The patient experienced a good postoperative course and was discharged on postoperative day 6. The pathological examination revealed a typical nesting pattern (zellballen pattern), which is characteristic of a noradrenaline-producing pheochromocytoma [2]. The tumor cells had granular cytoplasm and were immunohistochemically positive for chromogranin A, and synaptophysin (Figure 3).

**Figure 4:**
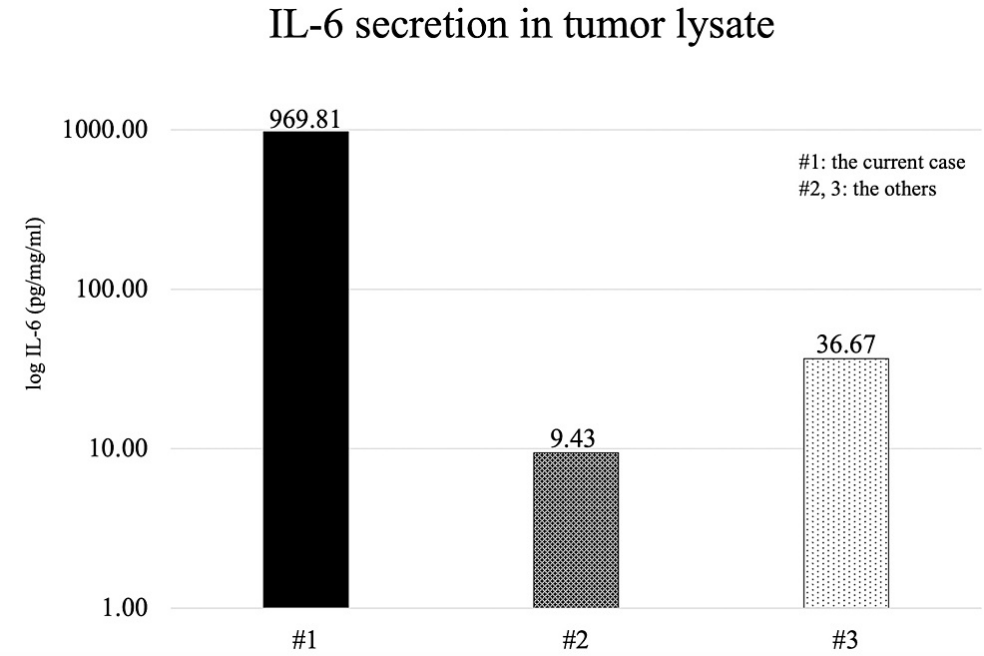
IL-6 secretion determined via enzyme-linked immunosorbent assay Enzyme-linked immunosorbent assay revealed increased IL-6 secretion in the current case. #1, the current case; #2, 3, the other cases.

The pathological diagnosis was pheochromocytoma with a grading of adrenal PPGL score of 2 (well-differentiated type). Using an enzyme-linked immunosorbent assay (ELISA) from tumor tissue lysates, we compared the IL-6 secretion of the current case (#1) with two other cases of pheochromocytoma that presented neither elevated serum IL-6 nor high fever (#2, 3). This patient exhibited more IL-6 secretion than that seen in other cases (Figure 4).

**Figure 5:**
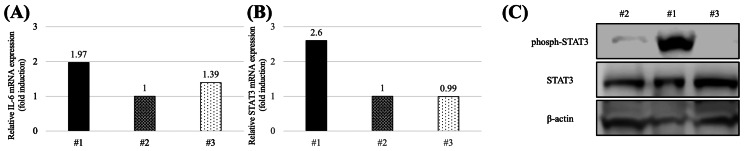
IL-6 and STAT3 expressions in resected specimens (A): The relative IL-6 mRNA level exceeded that in the other controls. (B): The relative STAT3 mRNA level exceeded that in the other controls. (C): The images show the immunoblot results and graphical quantification for STAT3 and β-actin. #1, this case; #2, 3, the other cases. The asterisk indicates a significant difference (p < 0.05). STAT, signal transducer and activator of transcription; mRNA: messenger RNA.

Thus, it was suggested that IL-6 was produced directly from the tumor tissue. Additionally, total RNA and protein were extracted from the resected tumors, and quantitative real-time PCR and immunoblot assays were performed. Both IL-6 mRNA and phosphorylated signal transducer and activator of transcription 3 (phospho-STAT3) mRNA expression levels exceeded those of the other cases. Similarly, phospho-STAT3 protein expression was higher than in the other cases (Figure 5).

Thus, we surmised that IL-6 expression was potentiated through the IL-6/STAT3 signaling pathway. To date, the patient has continued to undergo imaging follow-up for her left adrenal tumor and has been placed under observation with no increase in tumor size. Genetic analysis was not performed considering that the patient did not provide consent.

## Discussion

We herein describe a case of IL-6-producing pheochromocytoma associated with vHL disease in a young woman who presented with high fever. Although alpha-blockers and NSAIDs provided no antipyretic relief in the current case, steroids proved to be effective. To date, some published reports involving patients with IL-6-producing pheochromocytoma have shown that alpha-blockers and NSAIDs promote antipyretic relief [6-9]. According to these reports, these drugs decreased IL-6 and CRP secretion and alleviated symptoms associated with IL-6 hypersecretion. Moreover, Shimizu et al. reported that naproxen, indomethacin, and lipopolysaccharide directly suppressed IL-6 production in tumor cells collected from patients with IL-6-producing pheochromocytoma [6]. Similarly, another study reported that naproxen affects the posttranslational modification and secretory process of the IL-6 protein in experiments using human astrocytoma cell lines [10]. However, certain reports have demonstrated that alpha-blockers and NSAIDs were ineffective in reducing symptoms, similar to the current case [11,12]. It remains unclear which cases would not benefit from them. These drugs appear to be ineffective in some cases despite low IL-6 levels but effective in others despite high IL-6 levels. Further, efficacy and catecholamine or metanephrine levels were not significantly associated. 

The origin of IL-6 hypersecretion in IL-6-producing pheochromocytomas has been debated extensively. One hypothesis indicates that catecholamines, which are oversecreted by tumor cells, stimulate other immunocompetent cells and indirectly oversecrete IL-6 [5], whereas another hypothesis implies that tumor cells directly oversecrete IL-6 [13]. Our study showed that IL-6-producing tumor tissue lysates had increased IL-6 secretion compared to the other cases determined through ELISA. Furthermore, our quantitative real-time PCR and immunoblot findings also showed that IL-6 mRNA and protein expression were upregulated via STAT3 phosphorylation. If IL-6 hypersecretion were solely attributable to indirect factors, all IL-6-producing tumors would have exhibited abnormally high levels of catecholamines. However, previous reports have described IL-6-producing pheochromocytomas with normal catecholamine levels [8,11,12]. Based on these results, we concluded that tumor cells themselves overproduced IL-6 in this case. 

Recent advances in genetic analysis techniques have revealed, to date, more than 20 PPGL-causing genes. Conventionally, 10% of PPGLs used to be considered hereditary or familial; however, this concept has changed dramatically [14,15]. Today, approximately 40% of PPGL patients have variants in the germline [16]. Nonetheless, no reports have yet covered causative genes specific to IL-6-producing pheochromocytomas. One reason for this could be the many cases of IL-6-producing pheochromocytomas reported to date that have not been genetically analyzed. Recently, Toyoda et al. reported that tumor tissues from IL-6-producing pheochromocytoma were succinate dehydrogenase subunit B (SDHB)-positive on immunostaining, suggesting a mutation in the SDHB gene [7]. In addition, they demonstrated somatic mutations in vHL disease through comprehensive genome profiling and speculated that overproduction of IL-6 occurred via the activation of the Src-Lin28 pathway. However, given that immunostaining of tumor tissue had come back negative for Lin28, they stated that other mechanisms may be responsible for IL-6 overproduction. 

Our patient was diagnosed with vHL disease due to her clear family history and the presence of one typical tumor lesion according to the guideline [17]. This has been the first case report of an IL-6-producing pheochromocytoma associated with vHL disease. vHL disease had initially been inferred to have a gene localization on the short arm of chromosome 3 based on linkage analysis of the family tree in 1988. Moreover, in 1993, a group led by the National Cancer Institute (NCI) identified the gene responsible for vHL disease [18]. The percentage of families that do not develop pheochromocytoma can reach as high as 80% and are referred to as type 1 families. vHL disease type 1 results from mutations that cause severe protein activity defects, such as frameshift or nonsense mutations. In contrast, families that develop pheochromocytoma are called type 2 families, which are subdivided according to the presence or absence of renal cancer. Most vHL disease type 2 cases have missense mutations [19,20]. In line with this, the present case can be categorized as type 2. Nonetheless, no study has yet reported on the presence of IL-6 overproduction and genetic mutations in vHL.

## Conclusions

In conclusion, this case report underscores the rarity of IL-6-producing pheochromocytoma associated with vHL disease presenting with a protracted fever in a young woman. This study provides insights into the debated origin of IL-6 hypersecretion, emphasizing the direct action of the tumor. Furthermore, we surmised that IL-6 expression was potentiated through the IL-6/STAT3 signaling pathway. Further genetic analysis and exploration of the underlying mechanisms are warranted for a comprehensive understanding of this rare condition.
